# Effects of Metformin on Reproductive, Endocrine, and Metabolic Characteristics of Female Offspring in a Rat Model of Letrozole-Induced Polycystic Ovarian Syndrome With Insulin Resistance

**DOI:** 10.3389/fendo.2021.701590

**Published:** 2021-08-13

**Authors:** Yidong Xie, Li Xiao, Shangwei Li

**Affiliations:** ^1^Department of Obstetrics and Gynecology, West China Second University Hospital of Sichuan University, Chengdu, China; ^2^Key Laboratory of Birth Defects and Related Diseases of Women and Children (Sichuan University), Ministry of Education, Chengdu, China

**Keywords:** polycystic ovary syndrome, metformin, letrozole, offspring, metabolism

## Abstract

The beneficial effects of metformin, especially its capacity to ameliorate insulin resistance (IR) in polycystic ovary syndrome (PCOS), explains why it is widely prescribed. However, its effect on the offspring of patients with PCOS remains uncertain. This study investigated the impact of metformin treatment on the first- and second-generation female offspring born to letrozole-induced PCOS-IR rats. Forty-five female Wistar rats were implanted with continuous-release letrozole pellets or placebo and treated with metformin or vehicle control. Rats exposed to letrozole showed PCOS-like reproductive, endocrine, and metabolic phenotypes in contrast to the controls. Metformin significantly decreased the risk of body weight gain and increased *INSR* expression in F1 female offspring in PCOS-IR rats, contributing to the improvement in obesity, hyperinsulinemia, and IR. Decreased *FSHR* expression and increased *LHCGR* expression were observed in F1 female rats of the PCOS-IR and PCOS-IR+Metformin groups, suggesting that *FSHR* and *LHCGR* dysfunction might promote the development of PCOS. Nevertheless, we found no significant differences in *INSR*, *FSHR*, and *LHCGR* expression or other PCOS phenotypes in F2 female offspring of PCOS-IR rats. These findings indicated widespread reproductive, endocrine, and metabolic changes in the PCOS-IR rat model, but the PCOS phenotypes could not be stably inherited by the next generations. Metformin might have contributed to the improvement in obesity, hyperinsulinemia, and IR in F1 female offspring. The results of this study could be used as a theoretical basis in support of using metformin in the treatment of PCOS-IR patients.

## Introduction

Polycystic ovary syndrome (PCOS), a heterogenic disorder, affects 8–13% of reproductive-aged women; it is associated with reproductive and metabolic disorders, including hyperandrogenism, luteinizing hormone (LH) hypersecretion, infertility, polycystic ovaries, and insulin resistance (IR), with an increased risk of cardiovascular disease and type 2 diabetes (1). The etiology and pathogenesis of this multifactorial disease have not yet been completely identified. Based on familial clustering, genetic factors play a key role in the mechanism leading to this syndrome (2). Epigenetic changes during fetal life and lifestyle, hormone imbalances, and environmental factors might also contribute to the development and manifestation of PCOS (3, 4). Infertility has dominated clinical research involving PCOS patients, providing substantial evidence for an increased prevalence of pregnancy complications and less favorable pregnancy outcomes in women with PCOS, including preterm delivery, gestational diabetes, and hypertensive disorders (5, 6). Infants born to women with PCOS are also predisposed to adverse health outcomes. Accumulating evidence indicates that offspring of mothers with PCOS are at a higher risk for preterm birth, perinatal mortality, congenital abnormalities, and increased hospitalization rate (6–8). Obesity, metabolic dysfunction, comorbidity, and pregnancy complications of women with PCOS are likely to result in a suboptimal intrauterine environment that could have a detrimental impact on the health of infants and prepubertal children and contribute to an increased risk of developing PCOS in female offspring (9, 10). PCOS has been reported in 35% of premenopausal mothers of patients with PCOS (11). The underlying pathophysiological mechanisms of pregnancy complications and their association with the offspring’s health remain unclear.

Given the ethical limitations of human studies, PCOS animal models provide a valuable tool to study the developmental origin, pathogenesis, mechanisms, and long-term metabolic and endocrinologic impacts of PCOS, aid in detecting therapeutic strategies, and elucidate how the early life environment influences the offspring characteristics in later life. Various strategies have been used to induce PCOS rat models, including exposure to androgens, estrogens, antiprogesterone agents, constant light, and genetic modifications, during the critical period of pre/postnatal life (12). These models have some features of PCOS, but few have both metabolic and endocrine imbalances. Letrozole, a non-steroidal aromatase inhibitor, blocks the conversion of androgen to estrogen and subsequently increases androgen levels. Female rats treated with letrozole show disrupted cyclicity, increased LH and testosterone (T) levels, anovulation, absence of corpus luteum, thickened theca cell layer, thin granulosa cell layer, and increased ovarian weight. Continuous administration of letrozole to female rats, starting before puberty, induces endocrine/reproductive and metabolic abnormalities similar to those observed in women with PCOS (13). Specifically, these rats were shown to have IR, increased subcutaneous fat mass, enlarged adipocytes in the subcutaneous and visceral adipose tissues, anovulation, polycystic ovarian morphology, increased LH secretion, decreased follicle-stimulating hormone (FSH) secretion, and high ovarian expression of *Cyp17a1* mRNA (13, 14). Therefore, letrozole-treated rats are appropriate for studying PCOS mechanisms, consequences, and treatment.

The reduced conception rates associated with PCOS might be related to hyperandrogenism, obesity, and IR. The prevalence of IR in the general human population is 10–25%; nevertheless, in women with PCOS, it is approximately 60–70% (15). The increased IR and compensatory high insulin concentrations (hyperinsulinemia) play important roles in the progression of PCOS (16). IR, a prominent feature but not a criterion of PCOS, affects most PCOS patients and plays a role in PCOS development. Over time, IR might lead to glucose intolerance, which occurs in 40% of women with PCOS after the age of 40 years, and half of these women develop diabetes within 6 years (17). Pathophysiologically, IR could be caused by a defect in the insulin signal transduction (18). Metformin is an insulin sensitizer predominantly used to treat type 2 diabetes (19, 20). It inhibits hepatic gluconeogenesis and reduces the action of glucagon, resulting in reduced circulating insulin and glucose levels. Metformin is known to exert its effects on the liver, adipose tissue, and ovaries. Several studies have reported anthropometric and endocrine differences in women with PCOS between those with and without IR (15). As IR and the resulting hyperinsulinemia are key metabolic features in women with PCOS, their amelioration through metformin could improve PCOS-associated symptoms and conception rates (21). Lovvik et al. found a decreased risk of late miscarriage and preterm birth in those who received metformin throughout pregnancy (22). However, little is known about the effect of metformin on the offspring of women with PCOS and associated IR (PCOS-IR).

This study aimed to investigate the impact of metformin on reproductive alterations and developmental, endocrine, and metabolic characteristics in the first- and second-generation female offspring born to letrozole-induced PCOS-IR rats.

## Materials and Methods

### Animals and Experimental Design

The study was approved by the Animal Ethics Committee of West China Second University Hospital of Sichuan University. Forty-five female Wistar rats (F0) were included in this study. The rats were purchased from Chengdu Dashuo Experimental Animals Limited Company (Chengdu, Sichuan, China) and housed five per cage under standard conditions (12:12 h light-dark cycle, 23 ± 2°C, and 55–65% humidity), with *ad libitum* access to food and tap water. At the age of 21 days, the F0 rats were randomly divided into a control group (*n* = 15) and a letrozole group (*n* = 30). At 21 days of age, the letrozole group rats were implanted with 90-day letrozole continuous-release pellets (Innovative Research, USA) containing 36 mg of letrozole (daily dose of 400 μg). The control rats were implanted with a placebo lacking bioactive molecules (Innovative Research). The rat body weight was recorded weekly after implantation. At 70 days of age, tail blood was obtained after an overnight fast to assess fasting insulin and fasting glucose levels. Rats were injected intraperitoneally with a bolus of 1 g/kg glucose in 0.9% NaCl. Blood glucose was assessed at 15, 30, 60, and 120 min post-injection. At 77 days of age, serum P, E2, T, FSH, and LH were assessed.

**Figure 1:**
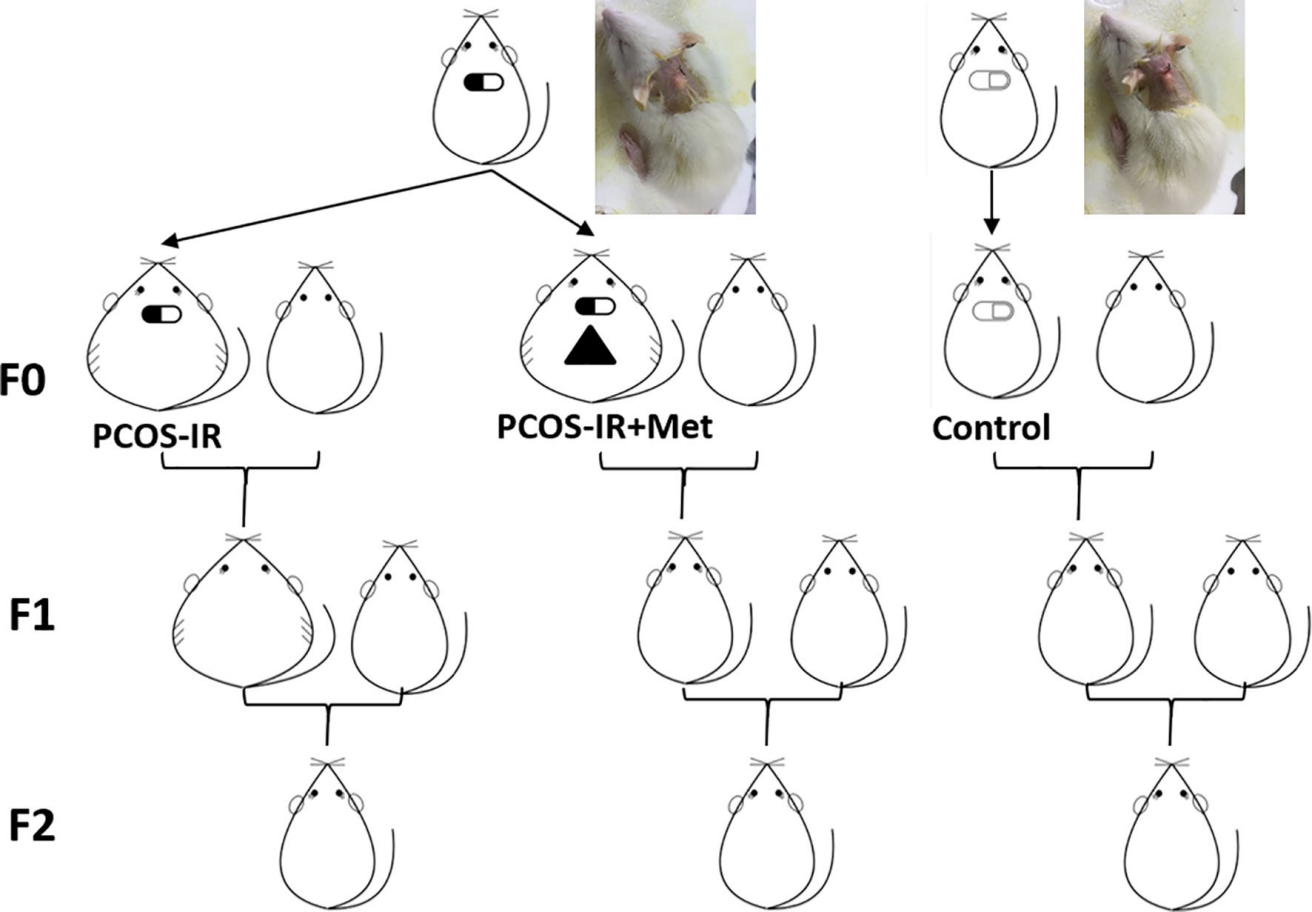
Schematic representation of experimental design and grouping. Female rats were implanted with letrozole-continuous-release pellets to establish PCOS-IR rat model. F0 female rats were randomly divided into PCOS-IR+Met, PCOS-IR, and Control groups and crossed with healthy male rats to obtain F1 offspring. F1 female offspring crossed with healthy male rat to obtain F2 offspring.

Two PCOS rats showed glucose impairment and were excluded from the following research. PCOS-IR model rats were defined as PCOS rats with IR but without glucose repair or diabetes. Twenty adult F0 female PCOS-IR rats were selected and randomly divided into PCOS-IR (*n* = 10) and PCOS-IR+Met (*n* = 10) groups. Ten of the control rats were selected as the control group. At 80 days of age, rats in the PCOS-IR+Met group were treated with metformin till pregnant. Rats in the PCOS-IR and control groups were not treated but were maintained under the same feeding and rearing environment. At 100 days of age, controlled ovarian stimulation was induced to promote offspring acquisition. Briefly, 20 IU of pregnant mare serum gonadotropin (Jianglai Biological, Shanghai, China) was injected intraperitoneally, followed 48 h later by intraperitoneal injection of 20 IU human chorionic gonadotropin (hCG; Jianglai Biological). Controlled ovarian stimulation was performed in all rats, which were then mated with healthy male Wistar rats. The number of pregnant rats in each group was recorded after collecting the vaginal plug. The number of successful deliveries, number of offspring, and offspring birth weight were recorded for each group. Ten female rats were randomly selected from each group of first generation (F1) and underwent 1:1 caged mating (to avoid inbreeding) with healthy male Wistar rats. The remaining female F1 rats were sacrificed for insulin receptor (*INSR*) expression testing in the pancreatic tissues and follicle-stimulating hormone receptor (*FSHR*) and luteinizing hormone/choriogonadotropin receptor (*LHCGR*) expression in the ovarian tissue. Upon the second generation (F2) birth, birth weight was recorded, and the female rats underwent the same tests as F1. The experimental design and grouping are shown in **Figure 1**.

### Vaginal Smears

The cyclicity stage was determined by microscopic analysis of the predominant cell type in vaginal smears obtained daily from the age of 11 weeks. The estrous cycle stage was determined by the main cell type in vaginal smears: proestrus, round nucleated epithelial cells; estrus, cornified squamous epithelial cells; metestrus, cornified squamous epithelial cells and leukocytes; and diestrus, nucleated epithelial cells and leukocytes.

### Tissue Sampling

Blood samples were obtained from the F0 rats at the age of 12 weeks (9 weeks after pellet implantation) for analyses of progesterone (P), 17β-estradiol (E2), T, FSH, and LH. All Serum samples were stored at –20°C pending analysis. The rats were decapitated, and ovaries were excised, fixed in neutral buffered 4% paraformaldehyde for 24 h, placed in 70% ethanol, dehydrated, and embedded in paraffin.

### Histomorphological Study

The paraformaldehyde-fixed and paraffin-embedded ovaries were longitudinally sectioned into 5 μm thick slices, mounted, and stained with hematoxylin and eosin (H&E) solution. The ovarian tissue morphology was evaluated under a light microscope by two researchers blinded to the origin of the sections.

### Endocrine Hormone Profile, Fasting Insulin Measurements, and Glucose Tolerance Test

Serum samples were obtained before sacrificing the rats, and stored at −20°C pending P, E2, T, FSH, and LH analyses. Endogenous hormone levels were measured using radioimmunoassay kits, following the manufacturer’s protocols. Fasting insulin and fasting glucose levels were measured with a radioimmunoassay kit, after overnight fasting of 8 h. For the intraperitoneal glucose tolerance test, the rats were injected intraperitoneally with a bolus of 1 g/kg glucose in 0.9% NaCl. Blood glucose was assessed at 15, 30, 60, and 120 min post-injection. All kits contained standard samples for quality control and were used following the manufacturers’ instructions. Thereafter, the area under curve of glucose against time was calculated. Additionally, fasting insulin and glucose values were used to determine the homeostasis model assessment of IR (HOMA-IR), which was calculated as fasting insulin (mIU/L) × fasting glucose (mmol/L)/22.5.

### RNA Isolation and Quantitative Real-Time PCR (RT-qPCR)

Total RNA was extracted from tissues using TRIzol reagent (Life Technologies Inc., Carlsbad, CA, USA) following the manufacturer’s protocol. The quality and purity of the extracted RNA were analyzed using a NanoVue Plus spectrophotometer (Healthcare Bio-Science AB, Uppsala, Sweden). cDNA was synthesized from purified total RNA using a PrimeScript RT reagent kit (TaKaRa Biotechnology Co. Ltd., Dalian, Liaoning, China). The primer sequences for *Insr*, *Fshr*, and *Lhcgr* (Sango Biotech, Shanghai, China) are listed in **Table 1**. RT-qPCR was performed using SYBR Green real-time PCR Master Mix (Toyobo, Osaka, Japan) and measured on an Applied Biosystems 7900 real-time PCR detection system (ABI, Foster City, CA, USA). The specificity of the PCR products was confirmed by the analysis of the dissociation curve. *GAPDH* was used as an internal control to normalize the target gene expression, and the relative expression was calculated according to the 2^–ΔΔCt^ formula. All experiments were repeated three times.

**Table 1:** Primer sequences of the qRT-PCR analysis.

Gene	Forward primer (5’-3’)	Reverse primer (5’-3’)
INSR	CCGTCGCTCCTATGCTCTGGTGTCA	TCGTGAGGTTGTGCTTGTTCCAGTCC
FSHR	CCTGGTCTCCTTGCTGGCATTCTTGG	TCGGTCGGAATCTCTGTCACCTTGCT
LHCGR	TCCAATGTGCTCCAGAACCAGATGCT	GCCACTCCCTGTCTGCCAGTCTATG
GAPDH	GAAGATCAAGATCATTGCTCCT	TACTCCTGCTTGCTGATCCA

### Western Blot Analysis

Total protein was collected from the pancreas and ovary using a radioimmunoprecipitation lysis buffer (P0013B, Beyotime Biotechnology, Shanghai, China), and the protein concentration was determined using a bicinchoninic acid assay kit (Beyotime Biotechnology). Equal amounts of proteins (50 µg) were separated by 10% sodium dodecyl sulfate-polyacrylamide gel electrophoresis and transferred to polyvinylidene fluoride membranes (Millipore, Billerica, MA, USA). The membranes were blocked for 1 h in 5% skim milk at room temperature and then incubated with primary and secondary antibodies. The following antibodies were used: rabbit anti-*INSR* (1:200; ab5500, Abcam, Cambridge, UK), rabbit anti-*FSHR* (1:200; GXP193389, GXP, USA), rabbit anti-*LHCGR* (1:200; GXP298459, GXP, USA), and mouse anti-β-actin (1:5,000, ab8226, Abcam) primary antibodies with which the membranes were incubated overnight at 4°C, and horseradish peroxidase-conjugated secondary anti-mouse/rabbit antibody for 1 h at room temperature. Protein bands were visualized using an enhanced chemiluminescence system (Millipore) and analyzed with ImageJ 2x (National Institutes of Health, Bethesda, MD, USA). Protein levels were normalized to the respective level of the β-actin internal controls.

### Statistical Analysis

Statistical analyses were performed using IBM SPSS Statistics for Windows, Version 19.0 (IBM Corp., Armonk, NY, USA) and Prism GraphPad (version 6.0, GraphPad Software, La Jolla, CA, USA). Continuous values are expressed as mean ± standard deviation (SD). The groups were compared by *t*-test, ANOVA with Bonferroni *post hoc* test, and chi-squared test as appropriate. For all comparisons, differences were considered statistically significant at *P* < 0.05.

## Results

### The Establishment of a PCOS-IR Rat Model

**Figure 2:**
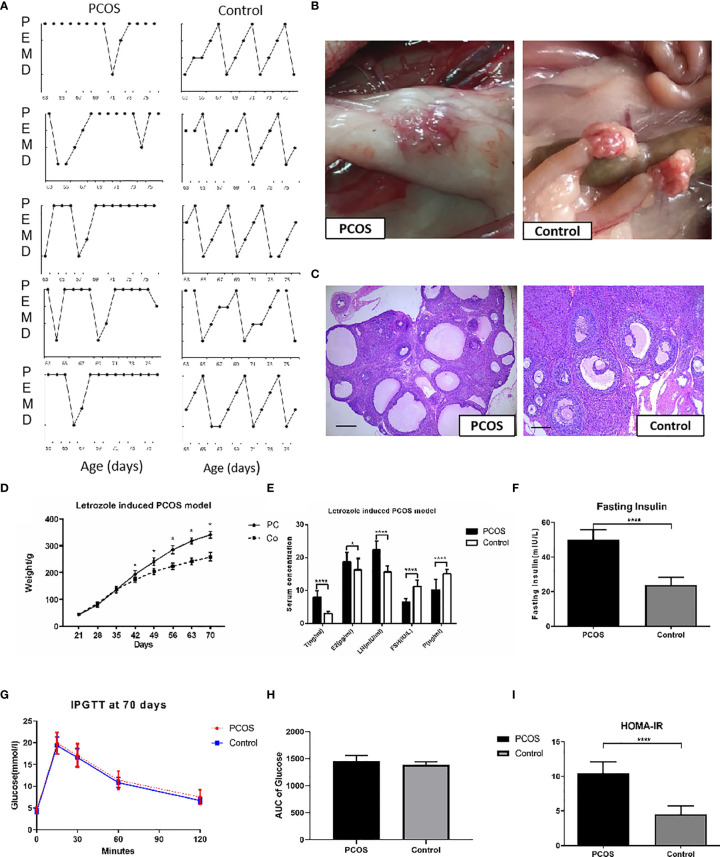
Establishment of PCOS rat model. **(A)** Disrupted estrus cycles of five sample cycles from each group were shown for each group. P, pro-estrus; E, estrus; M, metestrus; D, diestrus; **(B)** The gross ovaries. Ovaries from PCOS rats was significantly bigger than that from control group; **(C)** Histology of Ovaries. Ovaries from PCOS groups rats contained increased number of cystic follicles, granular cell layer thinning, and thickening of the theca cell layer. Ovaries from controls showed corpora lutea and mature follicles with healthy oocytes and thick layer of granulosa cells (scale bar = 200 μm). **(D)** Body weight changes of PCOS group. PCOS rats showed increased body weight; **(E)** Comparisons of T, E2, LH, FSH, and P in the rat serum from PCOS and control groups. **(F–I)** Comparison of glucose in Fasting Insulin, IPGTT, AUC of Glucose, and HOMA-IR from PCOS and control groups. The values were shown as the means ± SD. *p < 0.05 *vs.* Control, ****p < 0.0001 *vs.* Control, t-test.

The control group showed normal estrous cycles of 4–5 days. PCOS rats exposed to letrozole were acyclic, and vaginal smears showed that leukocytes were the predominant cell type, indicating pseudo-diestrus (**Figure 2A**). Ovaries from PCOS rats were enlarged, heavier, and surrounded by fatty tissue (**Figure 2B**). The micromorphology of the ovaries was observed following H&E staining. The rats treated with letrozole to induce PCOS showed a greater area of the largest follicles and more cystic follicles than the control rats, and their ovaries contained atretic antral follicles. We also observed granulosa cell layer thinning and thickening of the theca cell layer (**Figure 2C**). The body weight in the PCOS group increased remarkably 3 weeks after pellet implantation (**Figure 2D**). The serum sex hormone concentrations are shown in **Figure 2E**. The LH and T levels in the PCOS group were higher than those in the control, while E2, P, and FSH concentrations were significantly lower. Increased fasting insulin level and impaired IR, as determined by HOMA-IR in the letrozole-treated PCOS rats (**Figures 2F–I**), indicated abnormal endocrine and metabolic changes, although the areas under curve (AUC) of glucose concentration were comparable between groups. Two rats that presented glucose intolerance were excluded from subsequent analyses. Based on these results, we concluded that a PCOS-IR rat model was established.

### The Effects of Metformin Treatment on Pregnancy Outcomes in PCOS-IR Rats

In F0 rats, five rats were pregnant in the PCOS-IR group, seven in the PCOS-IR+Met group, and nine in the control group. The pregnancy rate was higher in the PCOS-IR+Met group than in the PCOS-IR group (**Table 2**). Two rats in the PCOS-IR group and one in the PCOS-IR+Met group died during late gestation due to placental abruption. Therefore, three rats in the PCOS-IR group, six in the PCOS-IR+Met group, and nine in the control group successfully delivered. The average number of offspring in the PCOS-IR and PCOS-IR-Met groups was significantly higher than that in the control group (**Table 2**). The three groups had similar female-to-male ratios among the F1 offspring. As for F1 generation rats, the pregnancy rates, live labors, and female-to-male ratios among those three groups were similar (**Table 2**).

**Table 2:** The impacts of treatment with metformin on pregnant outcomes.

		PCOS-IR	PCOS-IR+Met	Control
F0	Total dam (n)	10	10	10
	Number of Pregnancy (n)	5	7	9
	Number of Live labors (n)	3	6	9
	Number of Placenta abruption (n)	2	1	0
	Number of Offspring (n)	13.00±1.00^a^	14.67±1.21^a^	10.33+1.87
	Number of Female Offspring (n)	6.33±0.58	7.50±1.05	5.11±1.36
	Ratio of Female to Male	1.06±0.10	0.97±0.18	1.08±0.30
F1	Total dam (n)	10	10	10
	Number of Pregnancy (n)	8	8	9
	Number of Live labors (n)	8	8	9
	Number of Placenta abruption (n)	0	0	0
	Number of Offspring (n)	10.50±5.68	10.00±5.37	10.30±4.86
	Number of Female Offspring (n)	6.13±0.99	6.38±1.19	6.33±1.12
	Ratio of Female to Male	1.00±0.11	1.00±0.12	1.04±0.23

a P<0.05 versus Control.

### The Effects of Metformin on the Endocrine and Metabolic Changes in F1 Female Offspring

**Figure 3:**
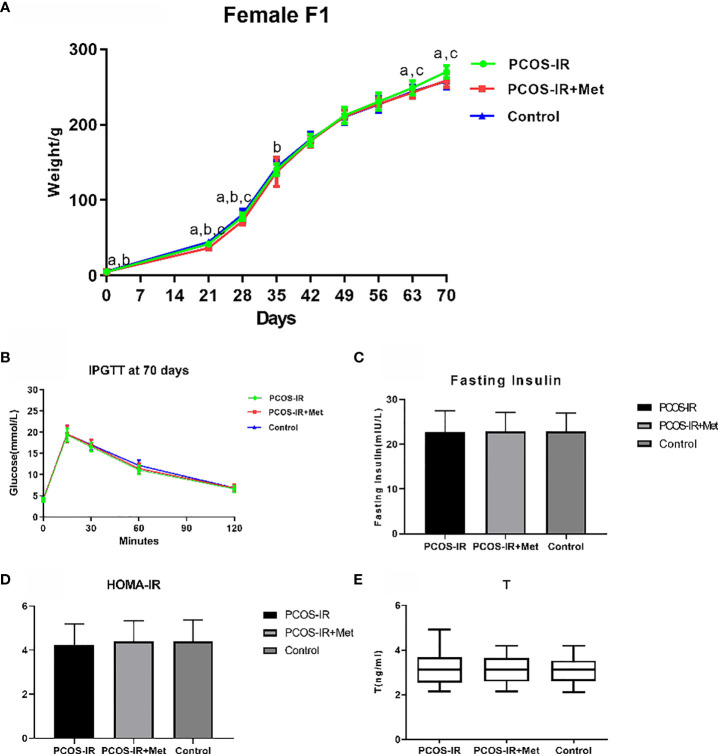
The effects of metformin treatment on the endocrine and metabolic alterations in female F1 offspring. **(A)** Body weight changes in female F1 offspring (PCOS-IR group, n = 19; PCOS-IR+Met group, n = 48; Control group, n = 46). a *P* < 0.05 PCOS-IR *vs.* Control, b *P* < 0.05 PCOS-IR+Met *vs.* Control, c *P* < 0.05 PCOS-IR *vs.* PCOS-IR+Met, ANOVA; **(B–E)** Glucose, fasting insulin, HOMA-IR, and T changes in female F1 offspring (PCOS-IR group, n = 19; PCOS-IR+Met group, n = 48; Control group, n = 46). There were no significant changes during those groups, ANOVA.

The offspring birth weight in the PCOS-IR+Met and PCOS-IR groups was lower than that in the control group. The F1 female rat body weight in the PCOS-IR group was significantly higher than those in the PCOS-IR+Met and control groups at the age of 63 and 70 days, once reaching sexual maturity. The body weights of the PCOS-IR+Met and control groups upon reaching sexual maturity were similar (**Figure 3A**). Glucometabolic index measured by the intraperitoneal glucose tolerance test, fasting insulin, HOMA-IR, and T levels were similar in F1 female rats of all three groups, both in the prepubertal period and once reaching sexual maturity (**Figures 3B–E**).

**Figure 4:**
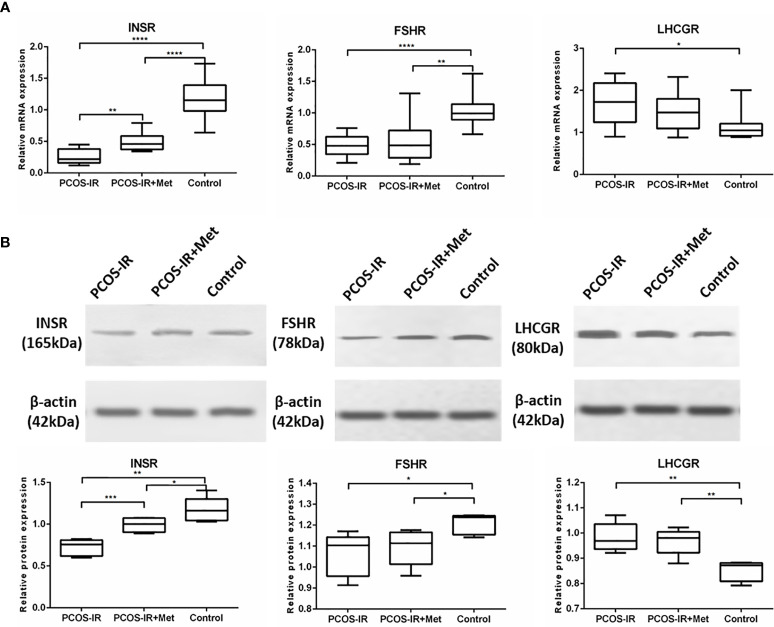
The effects of metformin on INSR, FSHR, and LHCGR expressions in female F1 offspring. **(A)** INSR mRNA expression in pancreas, FSHR and LHCGR mRNA expression in ovaries following treatment with metformin or placebo as measured by RT-qPCR (PCOS-IR group, n = 9; PCOS-IR+Met group, n = 10; Control group, n = 10). **(B)** INSR protein expression in pancreas, FSHR and LHCGR protein expression in ovaries following treatment with metformin or placebo as measured by Western Blotting (PCOS-IR group, n = 5; PCOS-IR+Met group, n = 5; Control group, n = 5). The values were shown as the means ± SD. *p < 0.05, **p < 0.01, ***p < 0.001, ****p < 0.0001.

We found that *INSR* mRNA expression in the pancreas of female F1 rats in the PCOS-IR group was significantly lower than that in the control group, while metformin increased *INSR* expression (**Figure 3A**). Ovarian *FSHR* expression in F1 females in the PCOS-IR and PCOS-IR+Met groups was significantly lower than that in the control group, with no difference between them (**Figure 4A**). The results also showed that *LHCGR* expression in PCOS-IR F1 female rats was significantly higher than that in the control group but similar to that in the PCOS-IR+Met group. *LHCGR* expression was also similar between the PCOS-IR+Met and control groups (**Figure 4A**). Western blot results suggested that LNSR protein expression in the pancreas and FSHR and LHCGR expression in the ovaries showed the same trend as the expression of their mRNA (**Figure 4B**).

Together, these findings suggested an elevated risk of PCOS development in the F1 female rats generated by mating females exposed to excessive levels of letrozole with healthy males. Metformin treatment reduced this risk.

### The Effects of Metformin on Endocrine and Metabolic Changes in PCOS-IR F2 Female Offspring

**Figure 5:**
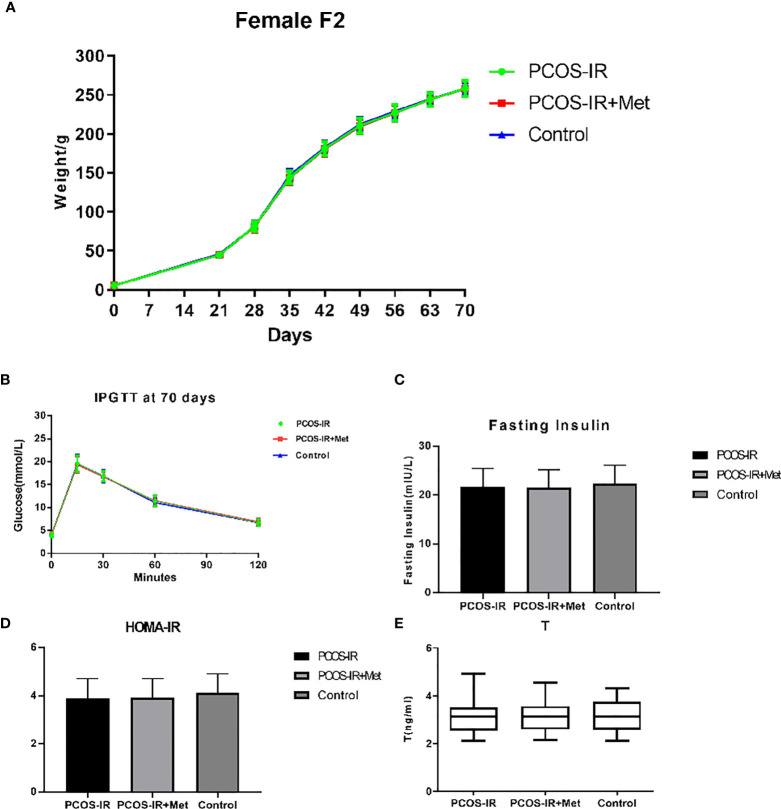
The effects of metformin treatment on the endocrine and metabolic alterations in female F2 offspring. **(A)** Body weight changes in female F2 offspring (PCOS-IR group, n = 49; PCOS-IR+Met group, n = 53, Control group, n = 47); **(B–E)** Glucose, fasting insulin, HOMA-IR, and T changes in female F2 offspring (PCOS-IR group, n = 49; PCOS-IR+Met group, n = 53; Control group, n = 47). There were no significant changes during those groups, ANOVA.

**Figure 6:**
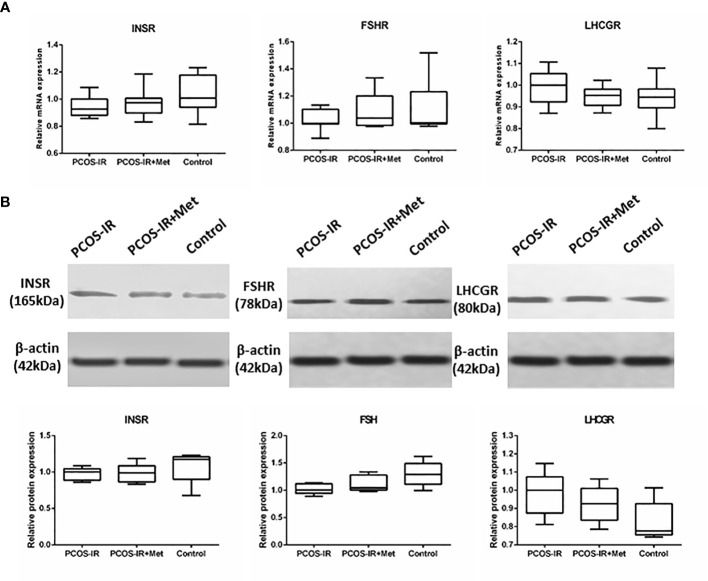
The effects of metformin on INSR, FSHR, and LHCGR expressions in female F2 offspring. **(A)** INSR mRNA expression in pancreas, FSHR and LHCGR mRNA expression in ovaries following treatment with metformin or placebo as measured by RT-qPCR (PCOS-IR group, n = 9; PCOS-IR+Met group, n = 10; Control group, n = 10). **(B)** INSR protein expression in pancreas, FSHR and LHCGR protein expression in ovaries following treatment with metformin or placebo as measured by Western Blotting (PCOS-IR group, n = 5; PCOS-IR+Met group, n = 5; Control group, n = 5). The values were shown as the means±SD. There were no significant changes during those groups.

We did not detect any significant difference in the body weight between the groups in the F2 generation. The glucometabolic indexes of F2 female rats were also similar in the three groups, both in the prepubertal period and once reaching sexual maturity, as were the glucose levels in the intraperitoneal glucose tolerance test, fasting insulin, HOMA-IR, and T levels (**Figure 5**). The mRNA and protein expression profiles of pancreatic INSR and ovarian FSHR and LHCGR in the F2 female rats were also similar among the three groups (**Figure 6**).

Overall, these results indicated that PCOS phenotypes were not stably inherited by the F2 generation after mating females with PCOS and healthy males.

## Discussion

Given the complexity of the human body and the ethical limitations imposed on human studies, it is difficult to investigate the effect of the prenatal environment on subsequent generations in humans. IR in PCOS patients is a trending field of reproductive and endocrinological research, as IR appears to be the key factor in its pathophysiology (23). Metformin treatment in PCOS patients requires further investigation; in particular, its effect on the offspring of women with PCOS-IR has rarely been reported. Continuous letrozole administration is an appropriate approach to replicating PCOS-like phenotypes in rodents. Rats exposed to letrozole in this study showed PCOS-like reproductive and metabolic phenotypes in contrast to the controls. Similar to a previous study (24), we established a PCOS-IR animal model using continuous letrozole administration through a slow-release implant. This model was used to further investigate the effects of metformin on the offspring of animals with PCOS-IR. We observed that pregnancy outcomes significantly improved after metformin treatment. F1 female offspring of PCOS-IR rats were at a high risk of developing obesity after sexual maturity; however, metformin treatment could considerably counteract this phenomenon. Genome-wide association studies have identified variants in 11 genomic regions (loci) as risk factors for PCOS; however, only *INSR*, *FSHR*, and *LHCHR*, which encode receptors for insulin, FSH, and LH/hCG, respectively, have clear functional relevance to the pathophysiology of PCOS (25, 26). We also investigated these PCOS candidate genes, *INSR*, *FSHR*, and *LHCHR*, showing significant alteration in their expression in F1 but not F2 female offspring of PCOS-IR rats treated with metformin. Together, these findings indicated widespread reproductive, metabolic, and endocrinologic changes in the letrozole-induced PCOS rat model. However, PCOS develops due to multiple factors, not only genetic ones.

A successful animal model was vital for the continuation of this study. In our study, letrozole-treated rats showed increased body weight and PCOS-like reproductive and metabolic phenotypes, including significant changes in serum T, E2, LH, FSH, and P, IR, widespread metabolic abnormalities, disrupted estrous cycles, and polycystic ovaries. Similar to Maliqueo et al. (13), we investigated glucose impairment or diabetes in the letrozole-induced PCOS rat model. Intraperitoneal glucose tolerance tests found no significant differences in glucose levels at 0, 15, 30, 60, and 120 min between the PCOS and control groups, indicating that the letrozole-treated rats had the PCOS phenotypes of obesity, altered hormone levels, and IR without developing type 2 diabetes or glucose intolerance. Abnormal insulin tolerance was observed in our study, but glucose levels remained normal. Glucose levels might remain normal in PCOS despite IR because of the compensatory increase in pancreatic β-cell insulin production, resulting in hyperinsulinemia (27). A successful PCOS-IR female rat model was induced, and the downregulation study that followed based on this model was effective and valuable.

The conception rate of PCOS-IR rats in this study has decreased, while metformin treatment improved it; the multiple pregnancy rate in the PCOS-IR groups was significantly higher than that in the control group and decreased after metformin treatment. Metformin taken during pregnancy increased the fetal concentration of sex hormone-binding globulin (28) and reduced the secretion of inflammatory cytokines by trophoblast cells *in vitro* (29). Patients with PCOS have an increased risk of pregnancy complications (30). In the present study, metformin was associated with low multiple pregnancy rates. A Cochrane review concluded that ovulation and pregnancy rates were higher in women with PCOS taking metformin (31). A recent study found that metformin treatment from the late first trimester until delivery in pregnant women with PCOS might reduce the risk of a late miscarriage and preterm birth, with no substantial change in serious adverse events in either the mothers or offspring (22). In the present study, we found two cases of placental abruption in the PCOS-IR rats. Palomba et al. (30) found a three- to fourfold increase in pregnancy-induced hypertension and preeclampsia, a threefold increase in gestational diabetes, and a twofold higher chance of premature delivery among patients with PCOS. Hyperandrogenism, obesity, IR, and other metabolic changes might result in an increased risk of obstetric and neonatal complications (30). Women with PCOS present placental inflammation, thrombosis, and infarction, villous immaturity, and nucleated fetal red blood cells during pregnancy (32), which might contribute to placental abruption in PCOS. Metabolic dysfunction in mothers with PCOS compromises the placental function of female fetuses with a genetic susceptibility to PCOS, promoting fetal hyperinsulinemia that leads to hyperandrogenism and altered folliculogenesis *in utero* (30, 33).

The data in the present study revealed that the body weight of sexually matured F1 female rats in the PCOS-IR group was higher than that in the PCOS-IR+Met and control groups, indicating that the F1 female offspring exposed to metformin during pregnancy demonstrated higher weight gain than offspring of mothers without drug treatment. No difference in body weight was detected in the F2 generation between the groups, demonstrating that inheritance of the obesity phenotype in the F2 generation was unstable in PCOS. It was reported that metformin treatment during pregnancy in patients with PCOS increased the risk of later overweight in offspring at the age of 4 years compared with placebo-exposed children (34); however, the clinical implications, body composition, and metabolic health of the children in their study were unknown. These aspects should be further investigated with long-term follow-up of the children. The T level increased after letrozole administration, as usually observed in these murine models (13, 35). Our data revealed that the glucometabolic indexes and serum T levels of prepubertal and pubertal F1 and F2 female rats were similar in all three groups.

Fetal nutritional and endocrine programming *in utero* might affect neuroendocrine systems with long-term health consequences, such as hypertension, hypercholesterolemia, and impaired glucose tolerance (36). Both genetic and early-life environmental factors in the uterus might contribute to PCOS development (37). The effect of IR in patients with PCOS on fetal growth and *in utero* programming, independent of either obesity or gestational diabetes, still needs to be clarified. Defects in the expression and/or activity of the insulin receptor might contribute to hyperinsulinemia in women with PCOS (15). We observed in this study a drastic decrease in the pancreatic expression of INSR in PCOS-IR F1 rats compared with the controls. The number of insulin receptors is a major determinant of cellular response to circulatory insulin. Any decrease in its expression significantly reduces insulin sensitivity. Functional insulin receptors are vital for insulin binding and signal transduction, and alterations in their expression markedly impair insulin binding and subsequent signaling pathways (38). The presence of hyperinsulinemia in women with PCOS-IR has been reported before, possibly due to some defects downstream of the insulin receptor (39, 40). Cellular IR in PCOS has been shown to involve a novel post-binding defect in insulin signal transduction. Treatment of IR with a diabetes drug such as metformin has become the mainstream therapy for women with PCOS (27). In the present study, metformin significantly increased INSR expression in PCOS-IR F1 rats, possibly contributing to improved hyperinsulinemia and IR, thus reducing PCOS-IR incidence in the F1 female rats. Nevertheless, after mating the PCOS-IR F1 females with normal male rats, the INSR expression in the F2 rats was similar to that in the controls, regardless of metformin treatment. However, metformin treatment significantly reduced the expression of the *Insr* gene in sexually matured F1 female rats, possibly contributing to the reduced incidence of PCOS. Metformin has been shown to confer protective effects on the pancreas in mice following exposure to fatty acid-induced stress and chronically elevated glucose levels (41). The effects of metformin on metabolic regulation in the human pancreas remain to be elucidated.

Metformin treatment in PCOS has been associated with the increased hepatic synthesis of sex hormone-binding globulin and decreased ovarian and adrenal androgens. FSH is an important endocrine hormone regulating ovarian function by binding to its specific receptor, FSHR. It stimulates follicular development *via* FSHR activation and induces granulosa cell proliferation. LH also plays a critical role in folliculogenesis. Studies have found significantly higher LHR and lower FSHR levels in patients with PCOS than in controls (42–45). This study also revealed alterations in FSHR and LHCGR expression levels in the F1 offspring of PCOS-IR rats. Our results showed that the ovarian expression of FSHR decreased and LHCGR increased in F1 rats of the PCOS-IR and PCOS-IR+Met groups, suggesting that FSHR and LHCGR dysfunction might promote the development of PCOS. Our findings differ from those of Rice et al. (46), who found a reduction in basal levels of *FSHR* mRNA following metformin treatment. The expression of FSHR and LHCGR was greatly improved with metformin treatment. The expression of PCOS-related INSR and FSHR in F2 offspring of PCOS-IR rats was still abnormal but did not differ from that in the F2 offspring of normal rats. PCOS might be a genetic disease, but genetic factors might not be the only reason for its development. Non-genetic factors could also play an important role in PCOS pathogenesis.

Nevertheless, this work is limited by being a preliminary-stage investigation. Further functional studies are needed. Clinical studies could be performed to observe the effect of metformin on the offspring of patients with PCOS and assess their risk of developing PCOS.

In conclusion, our findings indicated a higher risk of reproductive, metabolic, and endocrinologic abnormalities in PCOS-like model rats and their F1 offspring. Metformin treatment could improve pregnancy outcomes in these rats. The data relating to the F2 offspring showed them to be similar to the control and treatment groups. In women with PCOS, a possible combination of genetic, environmental, clinical, and biochemical factors is involved in this complex syndrome. Non-genetic factors play a key role in PCOS pathogenesis. However, metformin treatment significantly reduced the INSR expression level after sexual maturation in the F1 female rats, possibly contributing to the reduced PCOS incidence in this group. The study results could be used as a theoretical basis in support of metformin use to treat patients with PCOS-IR but without impaired glucose tolerance or type 2 diabetes mellitus.

## Data Availability Statement

The raw data supporting the conclusions of this article will be made available by the authors, without undue reservation.

## Ethics Statement

The animal study was reviewed and approved by the Animal Ethics Committee of West China Second University Hospital of Sichuan University.

## Author Contributions

All authors contributed to the study conception and design. YX and LX performed the experiments and acquired the data. YX, LX, and SL prepared the manuscript. All authors contributed to the article and approved the submitted version.

## Funding

This work was supported by a grant from National Natural Science Foundation of China (No.81671422) and a grant from National Key R&D Program of China (No.2016YFC100206).

## Conflict of Interest

The authors declare that the research was conducted in the absence of any commercial or financial relationships that could be construed as a potential conflict of interest.

## Publisher’s Note

All claims expressed in this article are solely those of the authors and do not necessarily represent those of their affiliated organizations, or those of the publisher, the editors and the reviewers. Any product that may be evaluated in this article, or claim that may be made by its manufacturer, is not guaranteed or endorsed by the publisher.
